# Connexin-43 enhances tumor suppressing activity of artesunate via gap junction-dependent as well as independent pathways in human breast cancer cells

**DOI:** 10.1038/s41598-017-08058-y

**Published:** 2017-08-08

**Authors:** Asif Raza, Archita Ghoshal, S. Chockalingam, Siddhartha Sankar Ghosh

**Affiliations:** 1 Department of Biosciences and Bioengineering, Indian Institute of Technology Guwahati, Guwahati-39, Assam India; 2 Centre for Nanotechnology, Indian Institute of Technology Guwahati, Guwahati-39, Assam India

## Abstract

The gap junction (GJ) protein connexin-43 (Cx43) is considered as a tumour suppressor protein for its role in reversing the phenotype of the cancer cells. In this study, we exploited the antitumor property of Cx43 in conjunction with the artesunate (ART), a plant-based active anti-malarial compound. The reactive oxygen species (ROS) generated by ART resulted in DNA damage, which in turn led to DNA damage response by activation of DNA damage repair proteins. GJ deficient MCF-7 cells transfected with Cx43 gene showed an increased sensitivity towards dose-dependent ART treatment and required a significantly lower dose of ART to attain its IC_50_, as compared to parental cells. This would ultimately result in reduced dose-dependent side effects of ART. The Co-culture experiments involving GJ intercellular communication (GJIC) deficient and GJIC enabled cells, established the transfer of ROS to the neighbouring cancer cells not exposed to ART. The ROS accumulated in the ART-treated cells induced the oxidative damage in neighbouring cells, leading to bystander cell death and inhibition of bystander cell proliferation. Thus, our study revealed that expression of Cx43 helped in reducing the dose-dependent cytotoxicity of ART as well as enhanced the bystander apoptosis of the neighbouring cells.

## Introduction

Gap junctions (GJs) are the fundamental entities for intercellular communications that contribute to cell differentiation, maintenance of normal cell growth, embryonic development, and tissue homeostasis^1, 2^. The first link between GJs and cancer was established when it was found that the rat hepatomas were devoid of GJIC^3^. Understanding the intricate association between GJs and cancer may shed considerable light on possible modes of cancer therapy. In this regard, a deeper comprehension of the role of connexins (Cxs), the building block of GJs, is desirable. Cxs are documented as tumour suppressor proteins as their re-expression into tumour cells decreases their tumorigenicity and reverses the transformed phenotypes of these tumour cells. Independent studies have shown tumour-suppressing effects of several Cxs (e.g. Cx26, Cx32, and Cx43)^4^. Among these, Cx43 has been studied extensively because of its widespread expression^5^. The tumour suppressive activity of Cx43 is not only limited to the exchange of specific molecules between normal cells and the tumour cells, but in some cases has also been found to be via GJ independent pathways^6–9^. In addition, GJ dependent mechanism of Cx43 helps in the spreading of prodrug (bystander effect)^10^ as well as small regulating signalling molecules in the neighbouring cells. Even though the transmission of stress signal from irradiated to non-irradiated cells was reported earlier, the responsible molecule(s) is yet to be identified^11^.

Due to the adverse side effects of the chemotherapeutic drugs, plant-based active anti-cancer compounds provide a better alternative. Artemisinin, a plant derivative, has been widely used as an antimalarial agent in the last few decades. However, its strong anticancer activity has been only explored recently^12–14^. ART is a semi-synthetic derivative of artemisinin, developed to overcome the pharmacokinetic limitations of artemisinin^15^. In addition, ART is recommended by World Health Organization due its good clinical efficacy and tolerability. Mode of action of ART involves the presence of endoperoxide bond, that is believed to be activated by reduced heme or ferrous iron (FeII), resulting in generation of cytotoxic ROS, which are strong alkylating agents^16, 17^. Also, the tumour cells are prone to ROS mediated damages as they exhibit downregulation of several antioxidant enzymes^18^. Normally, the dosage of ART required to have a cytotoxic response on tumour cells is much higher than those needed to exterminate malarial parasites. Consequently, an effective mode of co-therapy will be needed to counter the dose-dependent side effects of ART^19^.

Here, we have reported that overexpression of Cx43 sensitised the MCF7 cells towards the ART treatment. Also, the GJIC established between the neighbouring cells helped in the bystander killing of the cells after ART treatment. We also provided evidences in favour of the contribution of ROS generated by ART, in suppressing the growth of the neighbouring cells.

## Materials and Methods

### Reagents and chemicals

All the chemicals, reagents, and kits used in the following experiments were purchased from Sigma-Aldrich, unless otherwise mentioned.

### Cell culture and drug treatment

ACHN and MCF7 cells were procured from the National Centre for Cell Science (NCCS), Pune, India. Both the cell lines were grown in Dulbecco’s modified Eagle’s medium (DMEM high glucose), containing 10% fetal bovine serum (FBS) and 1% penicillin/streptomycin (100 U/ml; 0.1 mg/ml), in humidified air containing 5% CO_2_ at 37 °C. ART was made as a stock solution of 100 mM in dimethyl sulfoxide (DMSO). Carbenoxolone (CBX), a GJ inhibitor, was prepared as a 100 mM stock solution dissolved in water. Treatments with ART were performed (usually for 48 h) at varying concentrations.

### Cloning, transfection, and expression analysis

The total RNA was isolated from the Human renal adenocarcinoma cells (ACHN) using the GenElute Mammalian Total RNA Miniprep Kit. Furthermore, 1 μg of isolated RNA was used to generate cDNA library by using Verso cDNA Kit (Thermo Scientific, Waltham, MA, USA) following the manufacturer’s protocol. A semi-quantitative polymerase chain reaction (PCR) was performed with Cx43 forward and reverse primers having *EcoRI* and *ApaI* restriction sequence overhangs, respectively, using cDNA of the ACHN cells. The amplified Cx43 gene was cloned in pGEM-T Easy vector using gene-specific primers. Further, the gene was sub-cloned into pEGFP-N1, a mammalian expression vector. DNA sequence and sense insertion were confirmed by performing restriction digestion and sequencing.

Stable transfection of Cx43 was performed as described previously in ref. 20. The overexpression of Cx43 was assessed by semi-quantitative PCR and quantitative real-time PCR (qPCR; primer sequences provided in supplementary info). qPCR was performed using SYBR Green as reporter dye (Power SYBR Green PCR master mix, Applied Biosystem) in Rotor-Gene Q (Qiagen). β-actin was taken as an endogenous control. ΔΔCt method was used to calculate the relative Cx43 mRNA expression.

### Immunocytochemistry and laser scanning confocal microscopy

Cells were grown on a glass coverslip in 35-mm dishes until they reached sub-confluency. After washing with PBS (pH 7.4), cells were fixed with 4% paraformaldehyde (HiMedia Inc.), permeabilised using 0.5% v/v Triton X-100 and blocked using 1% w/v bovine serum albumin (BSA) in PBS at room temperature. Further, the cells were incubated overnight at 4 °C with 1:500 dilution of mouse anti-Cx43 monoclonal antibody. After three washes with PBS, the cells were then incubated with a fluorescein isothiocyanate (FITC)-conjugated anti-mouse antibody at a dilution of 1:200 in blocking buffer (PBS with 0.1% tween 20) for 1 h at room temperature. Coverslips were mounted on a glass slide and imaged using Zeiss LSM 880 laser scanning confocal microscope system (Zeiss Germany Ltd.).

### GJIC functional assay

The functionality of GJIC was assessed by dye transfer assay using calcein-AM (acetoxymethyl ester) and PKH26 dyes. Cells were grown in a 12-well plate to their confluency. Another population of cells were labelled with 5 µM calcein-AM (30 min at 37 °C) and 2 µM PKH26 (10 min at 25 °C) dyes. Dual stained cells were trypsinised and plated on top of the cells grown in a 12-well plate at a ratio of 1:50 (labelled: unlabelled). After 4 h of incubation, the cells were visualised using fluorescence microscope (Nikon Eclipse Ti-U, Tokyo, Japan).

### Assessment of cell viability

MCF7 and Cx43-MCF7 cells were plated in 96-well plate at a density of 7 × 10^3^ cells per well in serum-supplemented DMEM media and allowed to adhere for 24 h. The cell populations were exposed to increasing concentrations of ART (10 to 200 µM) or ART in combination with CBX (100 µM) or DMSO (control) or catalase (2,000 units/mL) for 48 h. After the onset of treatment period, the anti-cell viability activity of ART was assessed by the 3-(4,5-dimethylthiazol-2-yl)2,5-diphenyltetrazolium-bromide (MTT) assay (HiMedia, Mumbai, India). Fractional cell survival was assessed by taking absorbance at 570 nm (Bio-Rad, Hercules, CA, USA) and normalising the background measurement at 650 nm at each drug concentration. Each experiment was performed in triplicates and repeated at least four times. The viability of the untreated cells (or control group) was normalised at 100%.

### Apoptotic cell assessment

The healthy, early apoptotic, and late apoptotic cells were distinguished by using phycoerythrin (PE) Conjugated Annexin V/7-aminoactinomycin D (7-AAD), following treatment with 20 µM ART. MCF7 and Cx43-MCF7 cells were grown and treated with ART for 48 h. After the treatment duration was over, the cells were collected by trypsinisation, washed with PBS, and stained with PE Annexin V and 7-AAD, following the manufacturer’s protocol (BD Biosciences). Finally, the cells were analysed using CytoFLEX flow cytometer (Beckman Coulter). The morphology of apoptotic cell surface was also visualised under Field Emission Scanning Electron Microscope (FESEM; Carl-Zeiss). The data presented are the results from at least three independent experiments.

### Antibodies and western blotting

Anti-Skp2, anti-p27 ^Kip1^ and anti- p21^Cip1^ were procured from Sigma-Aldrich (Steinheim, Germany). Mouse anti- Cx43 was purchased from BD Transduction Laboratories (India). DNA damage antibody sampler kit (#9947) was bought from Cell Signalling Technology (Danvers, MA). Western blotting was performed as described previously^21^. CBX (100 µM) was added in the Cx43-MCF7 growing medium to assess GJ independent expression of Skp2, p27 ^Kip1^ and p21^Cip1^ proteins. The Western blot images are representative of three independent experiments.

### Cell proliferation assay

The proliferation of MCF7 and CX43-MCF7 cells were measured by flow cytometric analysis of CFSE (carboxyfluorescein diacetate, succinimidyl ester or CFDA-SE) loaded cells^22^. In brief, the MCF7 and Cx43-MCF7 cells were incubated with 5 µM of CFSE in serum-free medium for 30 min at 37 °C. Subsequently, the labelled cells were incubated in 6-well plate and after 24 h of seeding, cells were analysed in flow cytometer. This time point was considered as 0 h in analysis. After 72 h, the cells were again harvested and the data were aquired in CytoFLEX flow cytometer (Beckman Coulter) and analysed using FCS Express 6 software (De Novo software). The acquired histogram of CFSE fluorescence intensity were unimodal, therefore we used a simplified calculation for doubling time estimation. Assuming no cell death during the course of our study and all the cells were dividing, the mean doubling time of the cell population were estimated using T_d_ = T/log_2_ (F_0_)/(F_T_); here, F_0_ and F_T_ are geometric mean fluorescence intensity at 0 h and 72 h, respectively. We have taken T_d_ as 72 h.

### Cell cycle analysis

The cell cycle analysis of untreated and treated (36 h with 20 µM ART) MCF7 and Cx43-MCF7 cells were performed as described previously^20^ with little modifications. The cells were synchronised using serum-free medium for 24 h. After the completion of the treatment duration, cells were harvested, fixed and subjected to flow cytometric evaluation. The data were acquired using CytoFLEX flow cytometer (Beckman Coulter) at an excitation wavelength of 488 nm, and emission was recorded in PE-A channel. The data were then analysed using ModFit LT software (Verity Software House).

### Intracellular ROS detection

The level of intracellular ROS was estimated using 2′,7′–dichlorofluorescein diacetate (DCFH-DA) assay. Briefly, MCF7 and Cx43-MCF7 cells were cultured at a density of 5 × 10^4^ cells per well of a 6-well plate and incubated overnight for attachment. Thereafter, the cells were treated with 20 µM of ART with or without 500 µM of N-acetylcysteine (NAC) in the serum-enriched medium for 6 h. Subsequently, the cells were washed with 1X PBS and incubated with 1 μM of DCFH-DA for 30 min at 37 °C in serum-free media. After labelling with DCFH-DA, the cells were washed repeatedly with PBS and harvested for flow cytometric evaluation. The data were then acquired using CytoFLEX flow cytometer (Beckman Coulter) at an excitation wavelength of 488 nm, and emission was recorded in FITC-H channel. The data were analysed using WinList 3D 9.0.1 software (Verity Software House).

### Fluorescent dye based assessment of bystander effect

The fluorescein-based dye CFSE was used for cell labelling. Cx43-MCF7 cells were first treated with 20 µM of ART for 6 h at 37 °C in a 6-well plate (50% confluence). After the treatment period was completed, the ART containing medium was replaced with fresh medium containing 10% serum. Another population of MCF7 or Cx43-MCF7 cells was labelled with 5 µM of CFSE in serum-free medium for 30 min at 37 °C. After extensive washing, the CFSE-labelled cells were added to each well containing ART pre-treated Cx43-MCF7 cells. CBX (100 µM) was used to block GJIC and 500 μM of NAC was used to investigate the ﻿role of ROS. To assess the possible involvement of H_2_O_2_, cells were also pre incubated with 2,000 units/mL of catalase. The ratio of ART pre-treated cells to CFSE-labelled untreated cells was ~3:1. Untreated cells were cocultured with CFSE labelled cells as a negative control. After 48 h of being cocultured, the cells were exposed to propidium iodide (PI; 1 µg/ml) to assess cell death. The ART pre-treated cells showed red nuclei while the bystander CFSE labelled cells nuclei appeared yellow due to green fluorescent CFSE dye. Quantitation of yellow nuclei were performed by taking multiple images using the fluorescence inverted microscope (Nikon Eclipse Ti-U, Tokyo, Japan).

To assess the effect of ART on the growth rate and proliferation of the bystander cells, the CFSE labelled cells were cocultured in a 12-well plate with ART pre-treated Cx43-MCF7 cells. Coculture of CFSE labelled MCF7 or Cx43-MCF7 cells with untreated Cx43-MCF7 cells was taken as control. CFSE-labelled cells were counted using fluorescence microscope 4 h after plating (baseline) and 48 h after cocultured. Experiment was performed in triplicates and cells were counted in three separate areas of 0.4 mm^2^ per field in each well. Thus, the number of cells/mm^2^ (cell density) was calculated as the mean of nine separate measurements.

### Statistical analysis

Data points were obtained from at least three different set of experiments in triplicates and expressed as the mean ± SD (SD = standard deviation). To calculate the statistical significance of differences, the two-way analysis of variance (ANOVA) and Tukey’s post hoc test were used for pairwise comparisons. Data were analysed using Prism, version 5.01 (GraphPad Software Inc., San Diego, CA, USA). Statistically significant values were taken as *p < 0.05, **p < 0.01, ***p < 0.001 and ****p < 0.0001. Non-linear curve regression analysis was used to assess the IC_50_ value for ART cytotoxicity.

## Results

### Overexpression of Cx43 in MCF7 cells formed a functional GJIC

**Figure 1:**
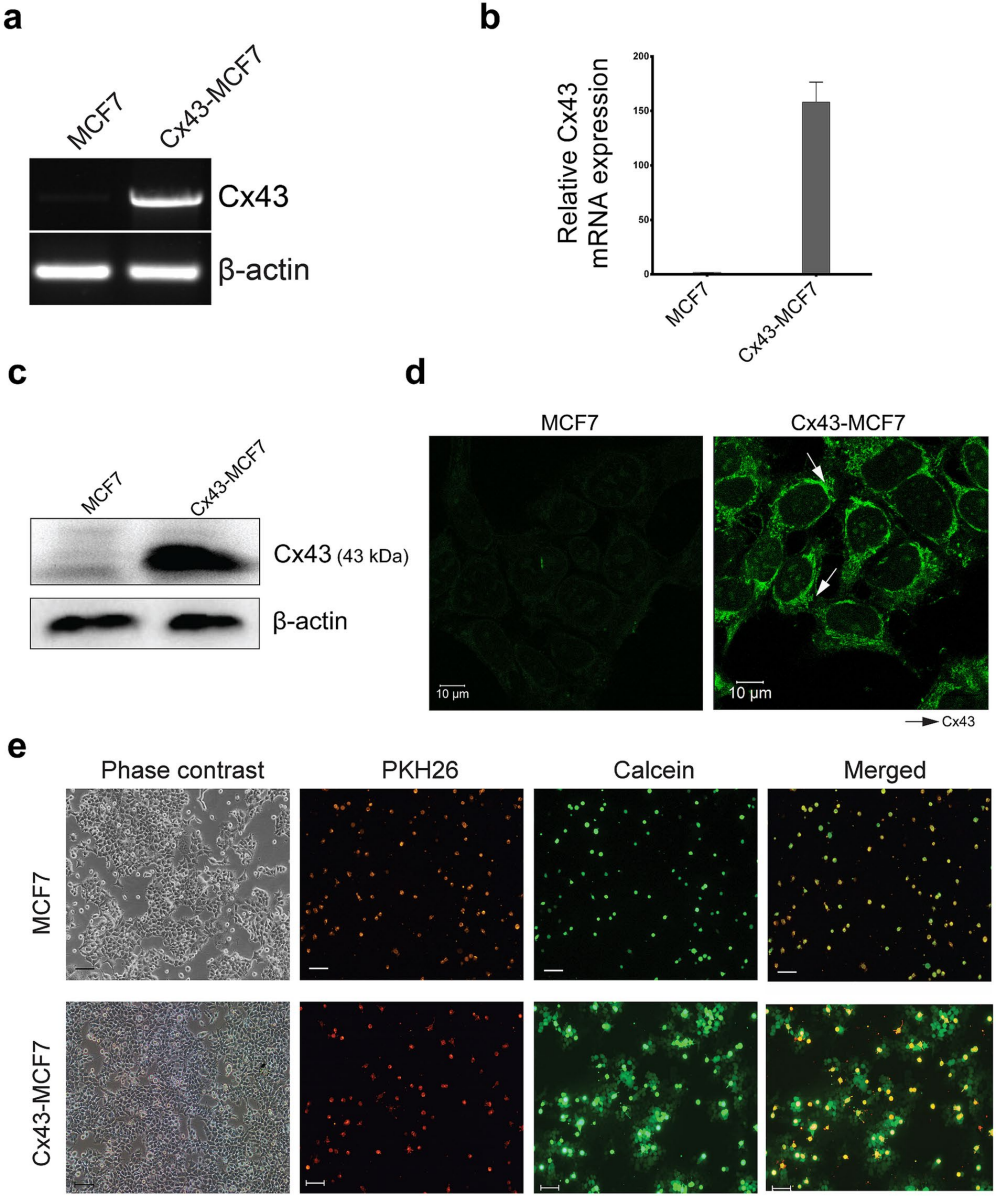
Cx43 overexpression and functional analysis of GJIC. (**a**) Expression level of Cx43 gene was initially examined by semi-quantitative PCR using cDNA library of MCF7 and Cx43 transfected MCF7 cells. The Cx43 gene band has been cropped and merged on top of the β-actin. (**b**) The comparative analysis of Cx43 mRNA expression in MCF7 and Cx43-MCF7 cells were analysed by using real-time PCR. The Cx43 mRNA expression in MCF7 cells was normalised to 1. (**c**) The Cx43 protein formation was then assessed by immunoblotting using anti-Cx43 antibody taking β-actin as endogenous control. (**d**) Represents the immunocytochemical analysis of Cx43 proteins in Cx43 transfected MCF7 cells revealed its presence at cell-cell contact points (arrows) and also in lesser extent inside cytoplasm. Bar = 10 µm. (**e**) Functionality of GJIC was determined by dye transfer assay using two different fluorescent probes viz. PKH26 and calcein AM. Bar = 100 µm.

The ACHN cells were screened for the endogenous expression of Cx43 (data not shown). The 1.2 kb long CDS of Cx43 encoded a protein of 382 amino acids with an estimated molecular mass of 43 kDa (GenBank accession no. CAG46461.1). The pEFGP-N1-Cx43 construct was then stably transfected into MCF7 cells. Figure 1a represents RT-PCR of cDNA library of Cx43-MCF7 lysate using Cx43 specific primers, amplified a gene having a size of 1.2 kb, which corresponds to the Cx43 gene. The relative expression analysis of Cx43 in MCF7 and Cx43-MCF7 cells showed the 157-fold increase in the expression of Cx43 in Cx43-MCF7 cells relative to the Cx43 expression in MCF-7 cells (Fig. 1b). The above PCR-based analysis confirmed the transfection and expression of Cx43 gene with the formation of Cx43 mRNA.

Expression of Cx43 protein in MCF7 and Cx43-MCF7 was examined by Western blotting with anti-Cx43 antibody using whole cell lysate (Fig. 1c). An intense immunoreactive band at 43 kDa corresponding to Cx43-MCF7 cell lysate suggested the overexpression of the Cx43 protein in the transfected cells. The band corresponding to the MCF7 cell lysate revealed basal expression of Cx43 protein in the cells. To examine the sub-cellular localisation of Cx43 more precisely, we subjected the Cx43-MCF7 cells to immunofluorescence reactions. In Cx43-MCF7 cells, patchy fluorescence with the granular appearance on cell surface suggested the varied localisation of the Cx43 proteins in the cytoplasm and the cell to cell junctions. However, the fluorescence density was highest in the areas of intercellular contact depicting the presence of Cx43 proteins, as shown in Fig. 1d.

In order to study the functionality of GJIC, MCF7 and Cx43-MCF7 cells were labelled with two different fluorescent dyes, PKH26 and calcein-AM. Due to its linkage with the cell membrane, PKH26 cannot be transferred from one cell to another. On the contrary, calcein, a green fluorescent dye generated from the calcein-AM when subjected to hydrolyse intracellularly by nonspecific esterases, can diffuse via GJ channels to the neighbouring cells as a marker for functional GJ coupling activity. Dual labelled MCF7 cells or Cx43-MCF7 cells were co-cultured with unlabelled Cx43-MCF7 cells; they settled on top of the unlabelled Cx43-MCF7 cells and got attached within 4 hour of plating. The extent of dye transfer after 4 h from MCF7 cells to Cx43-MCF7 cells was limited to one or no cell without any significant visual dye transfer. However, co-culture of dual-stained Cx43-MCF7 cells with the plated unlabelled Cx43-MCF7 cells showed a substantial increase in dye transfer among neighbouring cells, as about 15–30 unlabelled cells showed green fluorescence by sequential transfer of calcein from a single donor cell (Fig. 1e). PKH26 was retained by both the donor cells without any dye transfer.

### Overexpression of Cx43 in MCF7 cells sensitised it toward ART treatment

**Figure 2:**
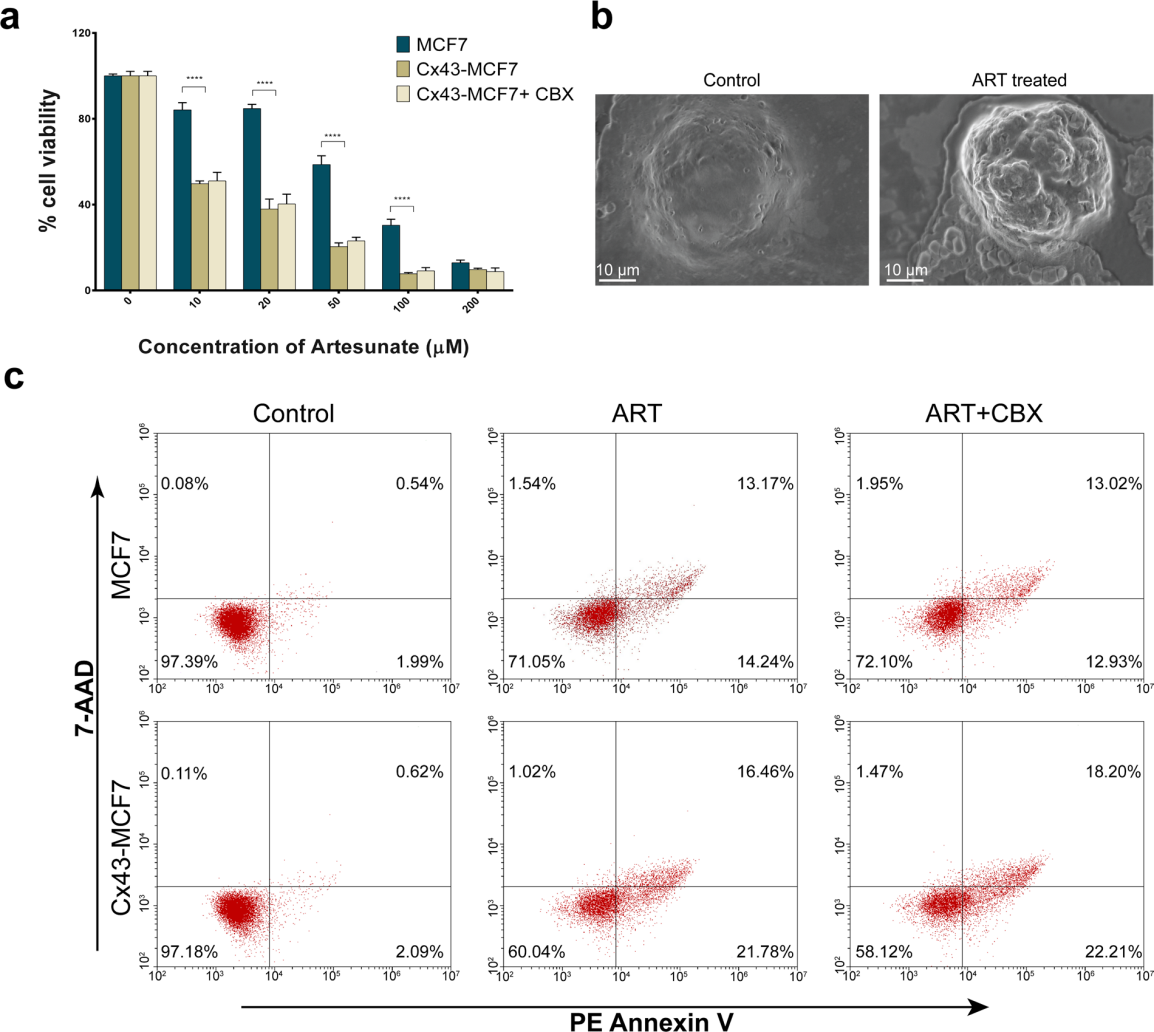
Cx43 overexpression in MCF7 cells enhanced the ART mediated cytotoxicity in dose dependent manner. (**a**) Both MCF7 and Cx43-MCF7 cells were subjected to treatment with increasing dose of ART or ART with 100 µM of CBX for 48 h. At the end of treatment duration, cell viability was assessed by MTT assay. (**b**) Represents the FESEM image of control and ART treated cell. Onset of apoptosis in the form of membrane blebbing was prominent on ART treated MCF7 cell. (**c**) Flow cytometry analysis of the cytotoxic effect of ART on MCF7 and Cx43-MCF7 cells determined by PE Annexin V and 7-AAD staining.

The sensitivity of MCF7 and Cx43-MCF7 cells towards the cytotoxicity of ART was examined by MTT assay. The MCF7 and Cx43-MCF7 cells were exposed to varying concentrations of ART for 48 h. Following treatment with ART, the Cx43 transfected cells showed a significant dose-dependent decrease in cell viability compared to untransfected MCF7 cells (Fig. 2a). Addition of GJ inhibitor, CBX, in combination with ART doses did not affect the dose-dependent decrease in cell viability of ART treated Cx43-MCF7 cells. Thus, the possibility of the involvement of GJs in the enhanced cytotoxicity of ART on Cx43-MCF7 cells was ruled out. The toxicity level (measured as IC_50_) induced by ART in Cx43-MCF7 was about 5-fold higher than that of untransfected MCF7 cells. In particular we observed that 58.98 ± 1.16 µM of ART concentration was required to reach its IC_50_ in MCF7 cells, while for Cx43-MCF7 cells, the IC_50_ concentration significantly reduced to only 11.22 ± 2.04 (mean ± S.D.; p < 0.05). The occurrence of apoptosis was further corroborated by FESEM imaging, in which membrane blebbing could be prominently visualised in the ART-treated Cx43-MCF7 cells as depicted in Fig. 2b.

In order to establish whether the enhanced sensitivity to ART induced by overexpression of Cx43 was associated with the progression of drug-induced apoptosis, we performed PE Annexin V/7-AAD FACS-based assay. MCF7 and Cx43-MCF7 cells were subjected to treatment with 20 µM ART for 48 h. After the treatment duration was completed, cells were stained with PE Annexin V and 7-AAD (which measure early and late apoptotic events, respectively) and analysed their fluorescence in flow cytometer (Fig. 2c). Untreated MCF7 and Cx43-MCF7 cells showed a low level of staining with either PE Annexin V or 7-AAD or both (2–4%). In contrast, ART-treated Cx43-MCF7 cells showed higher percentage of apoptotic cells (38.24%) when compared with ART treated MCF-7 cells (27.41%). Thus, the expression of Cx43 in MCF7 cells led to a substantial increase in the apoptotic cell population compared to untransfected MCF7 cells. Moreover, use of CBX with the ART did not reduce the extent of apoptosis, which, supported the concept of gap junction independent sensitisation of MCF7 cells after Cx43 transfection.

### GJ independent regulation of pro-apoptotic genes by Cx43 increased doubling time and induced G1 arrest in MCF7 cells

**Figure 3:**
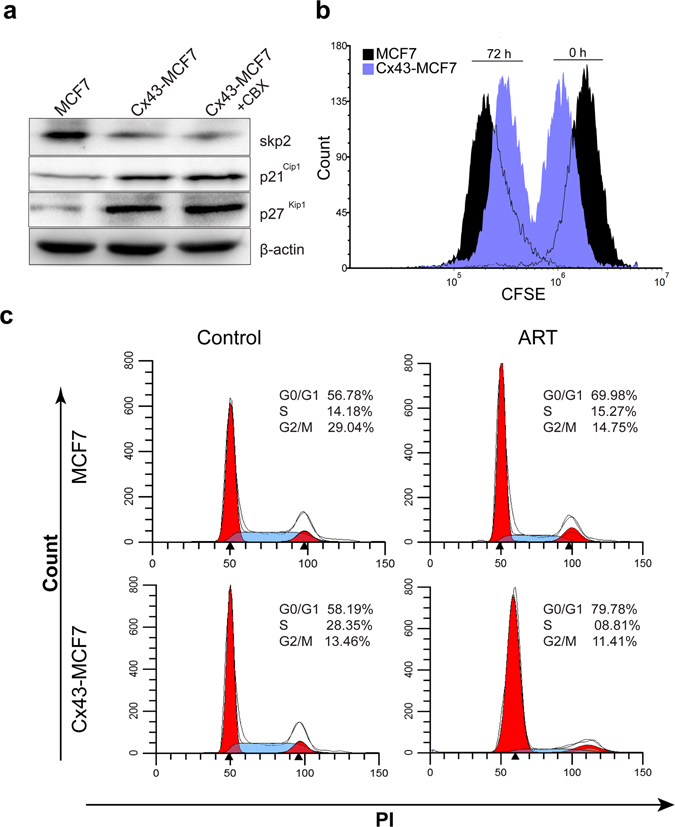
Anti-proliferative effect of Cx43 in MCF7 cells. (**a**) Regulation of anti-tumour proteins Skp2, p27 ^Kip1^, and p21^Cip1^ were determined after overexpression of Cx43 in MCF7 cells. The densitometric analysis was used to quantitate band intensity (supplementary Fig. 3). (**b**) CFSE fluorescence intensity histogram of MCF7 and Cx43-MCF7 in a representative experiment. The histogram moved leftward after 72 h of incubation, indicating dye dilution due to cell division. (**c**) Cell cycle profiling of MCF7 and Cx43-MCF7 cells after treatment with ART. Data were analysed using ModFit LT software.

To elucidate the mechanism by which Cx43 facilitate the enhancement of ART activity, we initially checked for the expression of anti-proliferative genes expression. We first ascertained the expression of Skp2 in MCF7 and Cx43-MCF7 cells, and found that the expression of Cx43 led to the downregulation of Skp2 protein (Fig. 3a). The downregulation of Skp2 further prevents the degradation of p27 ^Kip1^ and p21^Cip1^ proteins^6, 23^. Both p27 ^Kip1^ and p21^Cip1^ proteins are regarded as potent tumour suppressor proteins as their upregulation leads to the cell cycle regulation and cells become more responsive towards DNA damage response. Interestingly, addition of CBX in the Cx43-MCF7 growing medium (48 h) does not abrogated the expression of the above mentioned proteins, which inferred the GJ independent antitumor mechanism of Cx43.

The observation that Cx43 enhanced the expression of p27 ^Kip1^ and p21^Cip1^ protein, raised two possibilities. First, it is possible that the expression of these anti-tumour proteins leads to the reduction in the cells doubling time. Second, it is possible that these proteins help in the cell cycle arrest after DNA damage cause by ART treatment. To identify the delay in cell division, we pulse-labelled MCF7 and Cx43-MCF7 cells with CFSE. At two different time points, samples were collected and analysed in flow cytometer. Division of CFSE labelled cells led to a decrease in the dye fluorescence due to dye dilution (Fig. 3b). However, the unimodal peak shifting in Cx43 cells after 72 h was less when compared with MCF7 cells. This indicated that Cx43-MCF7 cells had lesser number of cell divisions in contrast to MCF7 cells in 72 h. After applying the equation as described in Materials and Methods section, we observed that the expression of Cx43 in MCF7 cells led to an increase in the doubling time of Cx43-MCF7 cells from 26 ± 2.75 h to 34 ± 3.12 h.

As we have observed the increase in the division time of Cx43-MCF7 cells, it is important to determine the alteration in cell cycle profile behind it. It was reported earlier that, increase in the expression of p27 ^Kip1^ and p21^Cip1^ proteins makes cell susceptible to G1 arrest^24, 25^. Based on these reports, we analysed the DNA content of the cell population for cell cycle analysis using PI. Figure 3c shows the flow cytometry data analysed in ModFit LT software, revealing a considerate amount of cell population present in G1 phase of cell cycle when treated with ART, as reported earlier^26^. However, the G1 population present in the treated Cx43-MCF7 cells (79.78%) were much higher when compared to treated MCF7 (69.98%) cells. Such difference in the G1 population suggesting that the Cx43-MCF7 cells were more susceptible to G1 arrest after treatment.

### DNA damaging ROS generated by ART in MCF7 cells initiated DNA damage response pathways in MCF7 cells

**Figure 4:**
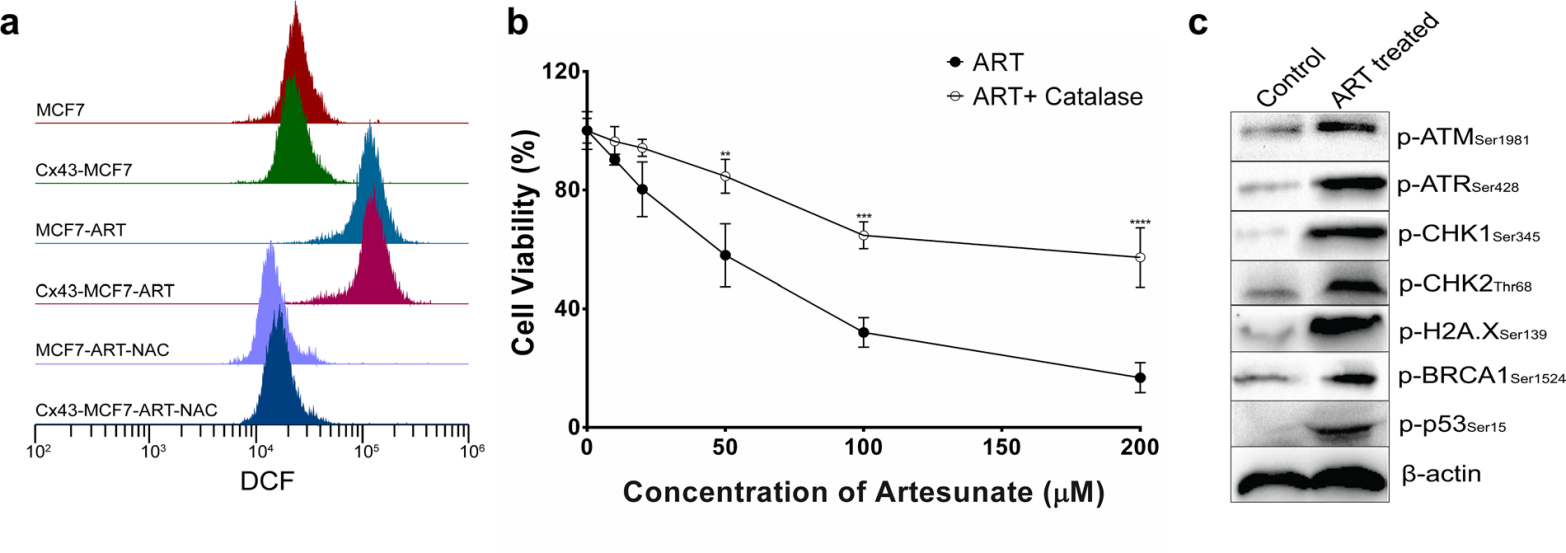
ROS generation and DNA damage analysis. (**a**) Generation of ROS in MCF7 and Cx43-MCF7 cells were assessed by measuring DCF fluorescence using flow cytometer. Untreated MCF7 and Cx43-MCF7 cells were taken as control and cells were treated in presence or absence of antioxidant NAC (5 mM). (**b**) Cell viability assay of MCF7 cells after treatment with ART in presence or absence of catalase. (**c**) ART induced DNA damage response in MCF7 cells upon exposure. Phosphorylation of ATM, ATR, Chk1, Chk2, histone H2A.X, BRCA1, and p53 was determined 8 h after the onset of treatment. β-actin was used as an endogenous control and to ensure equal proteins levels. The band intensity of the respective proteins were quantitated by densitometric analysis (supplementary Fig. 4).

To evaluate the role of ROS in ART-induced cell death, we determined the intracellular ROS levels using intracellular peroxide-dependent oxidation of DCFH-DA to form fluorescent DCF. Intracellular ROS clearly increased when both MCF7 and Cx43-MCF7 cells were treated with 20 µM of ART for 6 h at 37 °C. Fluorescent intensity of DCF is directly proportional to the level of ROS generated inside the cells. In Fig. 4a, the increase in the fluorescence of DCF in ART-treated MCF7 and Cx43-MCF7 cells compared to untreated MCF7 and Cx43-MCF7 cells clearly suggested the generation of the ROS in treated cells. Also, the fluorescence intensity of DCF was nearly the same in both treated MCF7 and Cx43-MCF7 cells, thus implying that there is no role of Cx43 in increasing the ROS generation after the treatment. Furthermore, to confirm that the increase in fluorescent intensity of DCF was due to ROS generation inside the cells, we used NAC. Addition NAC in the treatment medium reduced the DCF fluorescence intensity, thus confirming the production of ROS inside the cells brought about by ART treatment.

This cell viability based experiment was performed to investigate whether the extracellular generation of H_2_O_2_ might be involved in the cytotoxicity generated by ART. To test this possibility, we treated the cells with ART in presence or absence of catalase (2,000 units/mL) in the medium. The cell viability was examined by MTT assay (Fig. 4b). Catalase effectively prevented the cytotoxic effect of low dose of ART, as evidenced by the cell viability assay, but the data also suggested that at higher concentration of ART, the catalase did not prevent the complete cytotoxicity of ART. This might suggest the involvement of some other types of free radicals or processes in the ART-mediated cytotoxicity.

A previous report suggested that increased ROS formation by ART induces DNA damage response in the cells^27^. Initially, ROS mediated DNA damage was analysed by agarose gel electrophoresis. Genomic DNA analysis of apoptotic cell on agarose gel clearly showed the presence of fragmentation of the DNA in the treated population (see Supplementary fig. 2). To further confirm the apoptosis induced by DNA damage, we performed immunoblot for the proteins involved in major checkpoints in response to DNA damages (Fig. 4c). MCF7 cells were treated with 20 µM of ART for 24 h. At the end of the treatment period, the cells were lysed and subjected to Western blotting experiment. Increase in the expression of phospho-ATM (Ser1981) and phospho-ATR (Ser428) in ART-exposed cells indicated the DNA double-strand break and DNA single-strand break, respectively. Once the DNA damage response pathway was initiated by ATM and ATR, the downstream response proteins were also activated by phosphorylation. Chk1 and Chk2, the downstream protein kinases of ATM/ATR, get phosphorylated by ATM/ATR at Ser280 and Thr68, respectively. The histone H2A.X gets phosphorylated at Ser139 by ATM^28^. p53 and BRCA1 also get phosphorylated by upstream kinases. Phosphorylation of these signalling checkpoints proteins in response to DNA damage plays an important role in DNA damage checkpoint control, tumour suppression, and embryonic development.

### ART induced cytotoxic bystander effect via GJIC

**Figure 5:**
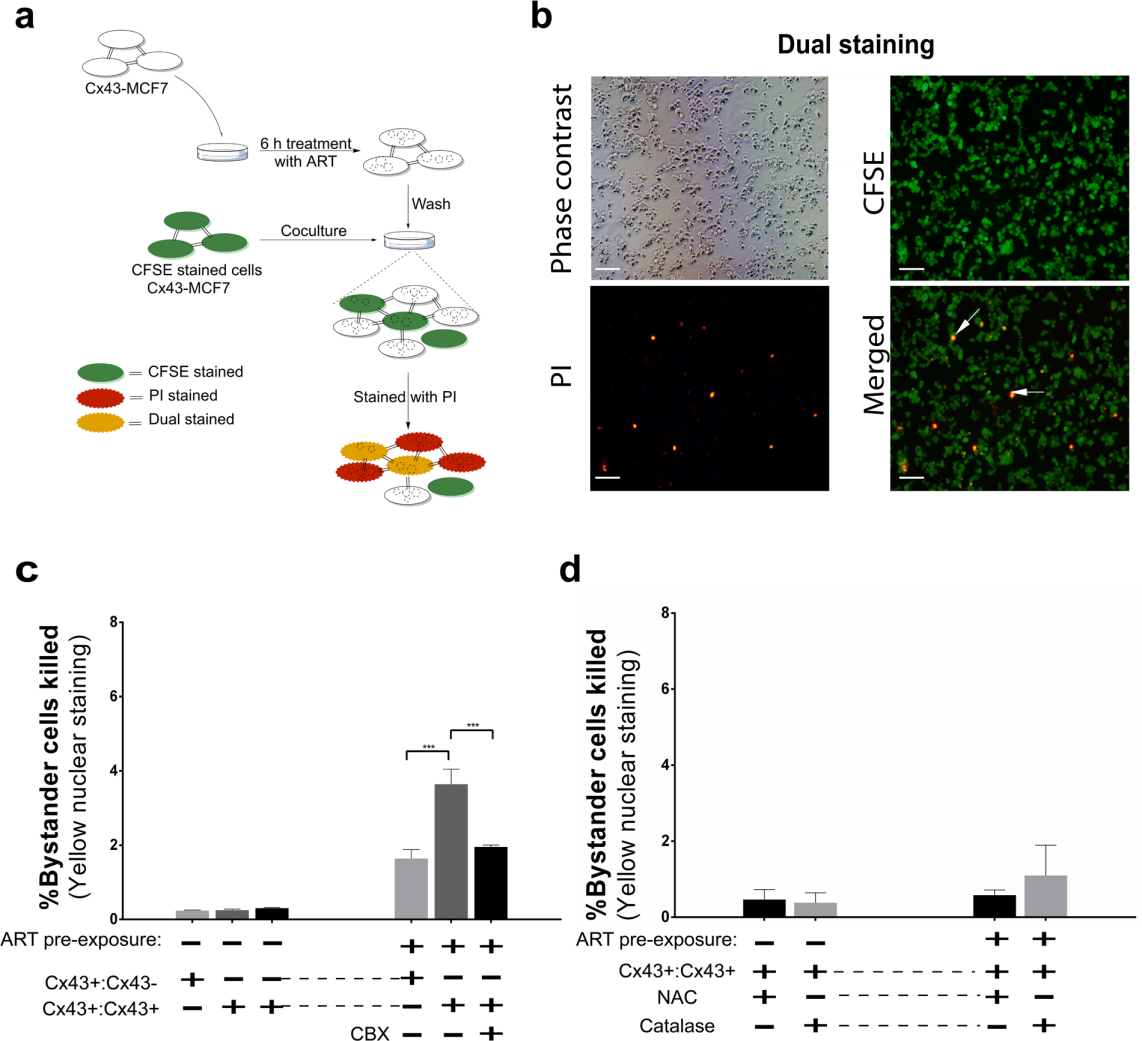
Induction of apoptosis in bystander cells after coculture with ART pre-treated cells. (**a**) Scheme of the experimental protocol used to delineate the bystander effect of ART. (**b**) After the onset of 48 h of coculture, the cells were stained with PI to examine the dead or membrane compromised cells. Nucleus of ART pre-treated dead cells appeared red while CFSE labelled membrane compromised cells showed yellow nucleus stain (arrow). Bar = 100 µm. (**c,d**) Quantitation of red and yellow nuclei after 48 h of coculture in ART pre-treated Cx43-MCF7 cells in presence or absence of CBX are presented. NAC and catalase were also used to examine the involvement of ROS in mediating bystander cell toxicity. The percentage of PI-labelled yellow nucleus cells counted from multiple fields (n = 6) was plotted.

We speculated that the ROS and DNA damage response induced by ART might spread among the neighbouring cells through GJ. To test the above possibility, we plated the Cx43-MCF7 cells to its sub-confluency and treated with 20 µM ART for 6 h. At the end of the treatment period, the drug-containing medium was repeatedly washed and replaced with the fresh medium. Another untreated population of MCF7 or Cx43-MCF7 cells were labelled with non-toxic green fluorescent dye CFSE and then after trypsinisation, co-cultured with washed pre-treated Cx43-MCF7 cells. Cells were stained with PI after 48 h of co-culture. Membrane compromised or dead cells that were ART-treated exhibited red nuclei whereas the CFSE labelled bystander cells showed yellow nuclei due to colabelling with green (CFSE) and red (PI) dyes (Fig. 5a and b). Figure 5c represents quantitative analyses of multiple images taken after 48 h of coculture revealing that the mean percentage of PI-labelled cells among the CFSE-labelled bystander cells was increased over 7-fold from 0.23% in untreated cocultured MCF7 cells to 1.64% in the treated cocultured MCF7 cells. Whereas, when Cx43-MCF7 cells were used instead of MCF7 cells for coculturing, the mean percentage of PI-labelled cells among the CFSE-labelled bystander cells were showed to have over 14.5-fold increase in the apoptotic cells. A significant drop of over 7.5-fold was observed in the mean percentage of cells with yellow nuclear staining among the CFSE-labelled cells when CBX was used in the media. Moreover, to investigate the involvement of the ROS in mediating the cytotoxic bystander effect, the ART pre-treated Cx43-MCF7 cells were cocultured with CFSE labelled Cx43-MCF7 cells in presence or absence of NAC or catalase in the medium. The reduction in the percentage of bystander cells in presence of NAC or catalase provided the evidence for the involvement of the ROS in mediating cytotoxic bystander effect of ART (Fig. 5d). Abolishment of the cytotoxic bystander effect of ART by using catalase in the medium strongly suggests the role of H_2_O_2_ in the process.

### Inhibition of bystander cell proliferation was enhanced by GJIC

**Figure 6:**
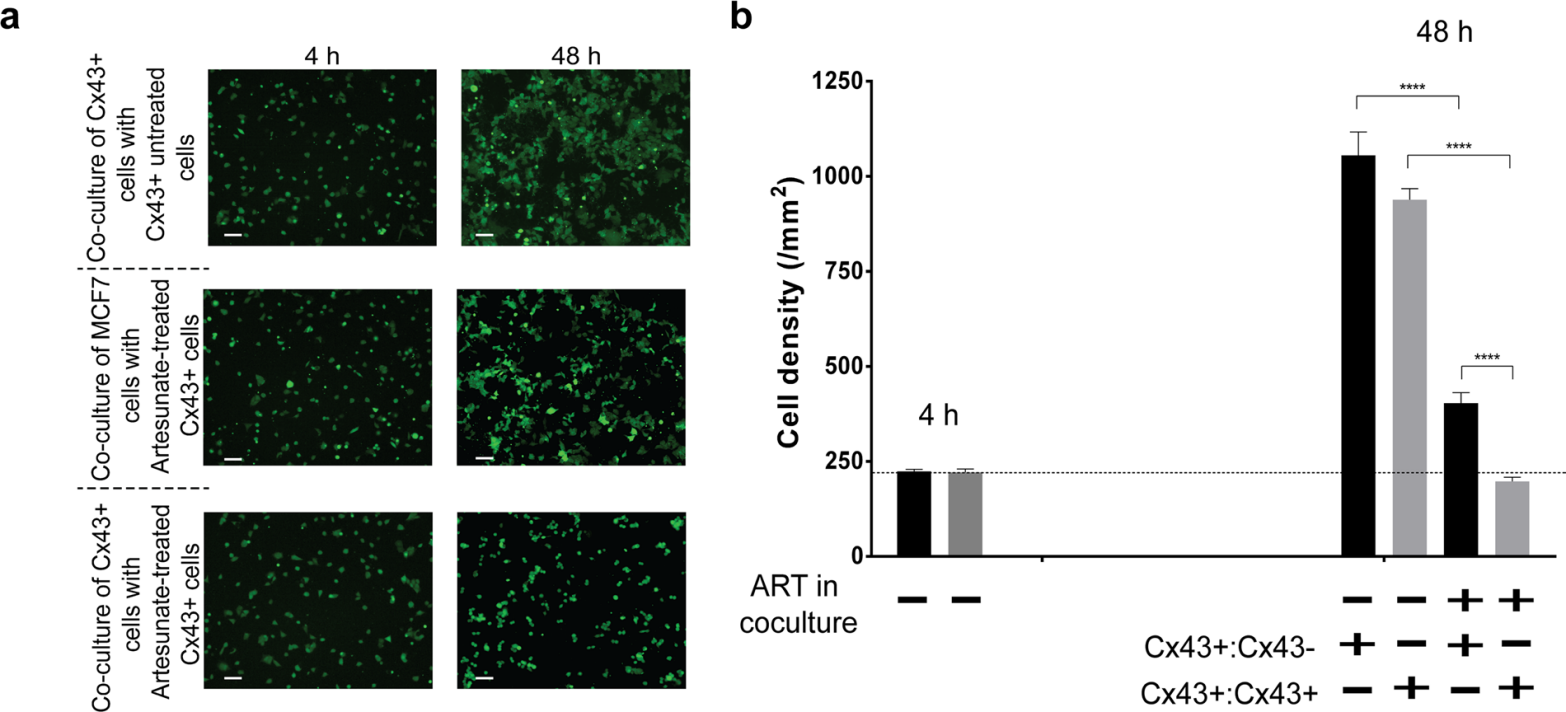
ART induced antiproliferative effect on bystander cells. MCF7 and Cx43-MCF7 cells labelled with CFSE were cocultured with unstained Cx43-MCF7 cells pre-treated with or without 20 µM of ART for 6 h. Green fluorescent cells were counted 4 h after plating and 48 h after coculture. (**a**) Image represented the density of CFSE labelled MCF7 or Cx43-MCF 7 cells after 4 h and 48 h of coculture with ART treated or not unlabelled Cx43-MCF7 cells. Bar = 100 µm. (**b**) Bystander cells in coculture were quantified in three independent experiments performed in triplicates.

Apart from lethal damage to the neighbouring cells, it is probable that ROS generated by ART might help in the decreasing the proliferation of the bystander cells. To test the above possibility, the MCF7 or Cx43-MCF7 cells were labelled with CFSE dye and plated on top of the Cx43-MCF7 cells, which were pre-treated with or without 20 µM of ART. Quantitative measurement of CFSE labelled cells (number of green cells/mm^2^) after 48 h of coculture provided the density of the cells grown. The density of CFSE labelled MCF7 or Cx43-MCF7 cells increased significantly from 223.67 ± 9.1 to 1055.3 ± 106.25 and 221.3 ± 14.57 to 938.67 ± 50.2 cells per mm^2^ after 48 h of coculture with untreated Cx43-MCF7 cells (control), respectively. Further, when the CFSE labelled MCF7 or Cx43-MCF7 cells were grown with ART pre-treated Cx43-MCF7 cells, there was a significant decrease or inhibition of the cell proliferation (Fig. 6a,b). After 48 h of coculture, the density of MCF7 cells grown with the pre-treated Cx43-MCF7 cells was found to be 403.3 ± 47.5 cells per mm^2^. Interestingly, the density of Cx43-MCF7 cells grown with the pre-treated Cx43-MCF7 cells was shown to have significant impact on the cell proliferation of the bystander cells as only 197.3 ± 18.5 green cells per mm^2^ were alive.

## Discussion

Lack of GJIC is more often found in various human tumours compared with adjacent normal tissues. Restoration of Cxs and /or GJIC in these tumour cells, particularly in early stage diseases, has been documented to have some unique therapeutic advantages in several *in vitro* and *in vivo* models^29^. In the present study, we exploited the tumour-suppressing property of Cx43 overexpression via GJ dependent as well as GJ independent pathways. We have found that the enforced expression of Cx43 in MCF7 cells increased its sensitivity towards ART treatment. ART has been most widely used as an anti-malarial drug since decades. We used a GJ deficient cell line MCF7 for the study of the mode of action of ART after forced establishment of GJIC and assessed the ART cytotoxicity through GJ dependent as well as GJ independent pathways. Here we show, for the first time that overexpression of Cx43 in MCF7 cells increased its ART-based cytotoxicity independent of GJ, and also the established GJIC helped ART in causing apoptosis and arresting proliferation of neighbouring untreated cells.

The transfection of Cx43-pEGFP-N1 into MCF7 cells led to the establishment of a stable cell line expressing Cx43. After transfection, we first checked the level of expression of Cx43 mRNA and compared it with untransfected MCF7 cells, we found that the mRNA of Cx43 was upregulated in Cx43-MCF7 cells, when compared with MCF7 cells alone. Consequently, the transcribed mRNA of Cx43 was translated into Cx43 protein, which was confirmed by Western blotting. The transfection of Cx43-pEGFP-N1 in MCF7 led to the overexpression of Cx43 proteins inside the cells. These proteins mostly localised on the surface and on the cell to cell contact formed a functional GJ. Cx43 proteins present on the point of cell-cell contact, formed a functional GJ, which established an active GJIC between neighbouring cells. The spread of calcein dye from stained Cx43-MCF7 cells to the unstained Cx43-MCF7 cells confirmed the functionality of GJIC.

In addition to GJIC, there are multiple pieces of evidence portraying the GJ-independent roles of Cx43 in the control of cell growth and the suppression of tumorigenicity. Huang *et al*.^30^ showed that the tumour suppressive effect of Cx43 was unrelated to the activity of GJIC. To delve deeper into this facet, we examined the combined effect of Cx43 overexpression in MCF7 cells and ART treatment. Recent experimental evidences suggest that anti-malarial ART may be a therapeutic alternative in highly aggressive cancers with rapid dissemination, without developing drug-based resistance^12, 31^. Although, a few reports of toxicities in cell lines and laboratory animals have raised concerns about the high doses and prolonged use of ART^19^, these findings have not been reported in humans taking numerous doses of artemisinin till date.

In our study, we found that the IC_50_ of ART treatment on MCF7 cells have significantly reduced when we overexpressed Cx43 in the cells. The dose required to reach IC_50_ in Cx43-MCF7 cells is ~5 fold less than that of MCF7 cells alone. Even though, when we used specific GJ inhibitor, CBX, it did not abrogate ART-based cytotoxicity, as examined by cell viability assay and PE Annexin V/7 AAD assay. The IC_50_ of ART for Cx43-MCF7 remained almost the same. The above result suggested that the Cx43-mediated enhancement of cytotoxicity is independent of GJ function.

Since we have delineated above that the overexpression of Cx43 in MCF7 cells enhances the cytotoxicity of ART, we further looked into the activation of underlying mechanism associated with it. Among the various cell cycle signalling proteins, downregulation of p27 ^Kip1^ and p21^Cip1^, an inhibitor of cyclin-dependent kinases, is linked with poor prognosis in many cancers. Both p27 ^Kip1^ and p21^Cip1^ promote cell cycle arrest in response to various stimuli^32, 33^. When we checked the expression of these proteins in Cx43-MCF7 cells, we found that there was a substantial decrease in the expression of Skp2 protein, while expression of both p27 ^Kip1^ and p21^Cip1^ was augmented, compared to the untransfected MCF7 cells. To rule out the possibility of the involvement of GJ in the expression levels of the above mentioned proteins, we also added CBX in the medium for 48 h to inhibit GJIC and then assessed the proteins expression. Interestingly, resulting data showed CBX had no effect on the protein expression. Thus, we can infer that Cx43 inhibited the expression of Skp2, which regulates the ubiquitination of p27 ^Kip1^ and p21^Cip1^, independent of GJIC. Therefore, we suggest that Cx43 may be involved in the sensitisation of Cx43-MCF7 cells towards the ART treatment by controlling the expression of Skp2, thereby increasing the level of tumour suppressor proteins, p27 ^Kip1^ and p21^Cip1^. Flow cytometric evaluation of the fluorescent intensity of CFSE labelled cells at different time intervals suggested an increase in the division time of Cx43-MCF7 cell population compared to the MCF cells. Based on the observation of increase in the expression of p27 Kip1 and p21Cip1, we were motivated to perform a cell cycle profiling of the treated cell population. The cell cycle profile suggested that the G1 arrest was more pronounced in Cx43-MCF7 cells. This might be one of the causes of the increase of ART sensitivity in Cx43-MCF7 cells.

It was postulated that iron-activated ART, which is considered as a prodrug, generates ROS such as hydroxyl radicals, superoxide anions, and highly alkylating carbon-centred free radicals^34, 35^. MCF7 cells provided an excellent model for our present study as it is devoid of Cx43 proteins and therefore GJIC. Also, the bioactivity of ART requires Fe(II), and it was previously reported that MCF7 cells express high concentration (5–15 times more than normal breast cells) of transferrin receptors on the cell surface and have a substantial amount of Fe(III) ion uptake into the cells^36^. When treated with ART, MCF7 and Cx43-MCF7 cells showed a high level of ROS production as depicted in Fig. 4a. Free radicals and ROS have been known to cause DNA damage in cells^28^. Intrigued by this, we sought to explore the DNA damage response pathway in Cx43-MCF7 cells after ART treatment. The MTT assay showed a considerable increase in cell viability when catalase was used with ART in medium, suggesting the role of H_2_O_2_ in mediating the bystander effect. After the observation of DNA fragmentation, we also started with the expression analyses of ATM/Chk2 and ATR/Chk1 pathways, which get activated in DNA damage responses. Overexpression of phospho-ATM and phospho-ATR after ART treatment indicated double-strand break and single-strand break of DNA, respectively. Once phosphorylated, the ATM and ATR phosphorylates a large number of downstream proteins^37^. Immunoblot data showed the overexpression of such proteins like phospho-Chk1 and phospho-Chk2. After it gets phosphorylated, Chk2 further activates p53 and BRCA1 proteins. BRCA1 helps in the double-strand break repair of DNA and cell cycle arrest in the S-phase, while p53 triggers cell cycle arrest in the G1-phase or cell death. We also found overexpression of phospho-Histone H2A.X, a novel biomarker of DNA damage response. ATM has been found to be the major protein that phosphorylates H2AX^38^.

**Figure 7:**
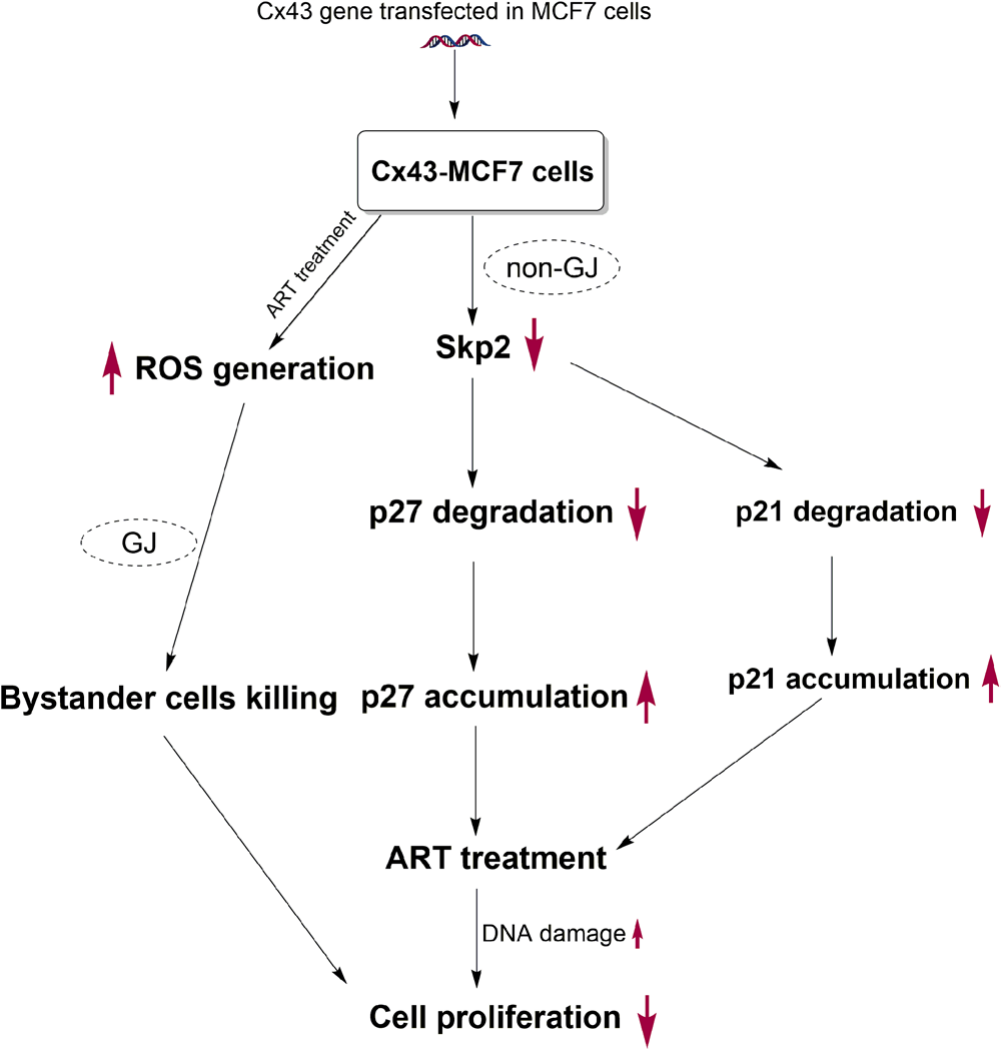
Schematic for the role of Cx43 in enhancing GJ dependent as well as GJ independent cytotoxicity of ART.

One of the striking features of the Cx43 expression is the formation of GJIC among cells. The basic strategy in GJIC-based therapies relied on the spreading of the cytotoxic molecules or signals in neighbouring cells, called ‘bystander effect’. Several studies have suggested a role of ART in the treated cancer cells, but whether ART treatment affects neighbouring cells remains elusive. Our study showed that even at the low clinically relevant concentration, ART induced cytotoxic bystander effect to the neighbouring cells. By using two different cocultured experiments we have illustrated that the GJIC helped ART in causing apoptosis as well as ceasing proliferation of the bystander cells. In the dual staining coculture experiment, we have found over 14.5-fold increase in the neighbouring untreated Cx43-MCF7 apoptotic cells. Although, we have found a significant percentage of apoptosis in untreated neighbouring MCF7 cells which do not form GJIC with treated Cx43-MCF7 cells, we speculate that it might be because of an extracellular release of ROS. The increase in the percentage of apoptotic cells in Cx43-MCF7 cells was dependent on the GJIC, as exhibited by the use of CBX in the medium, which brought about a significant drop in the percentage of apoptotic cells. It is worth noting that, as Cx43-MCF7 cells were more sensitive towards ART treatment, the percentage of apoptosis was more pronounced in these bystander cells compared to MCF7 cells alone. Moreover, the addition of NAC and catalase in the coculture medium abolished the bystander effect of ART. This suggested the possible involvement of H_2_O_2_ in mediating the cytotoxic bystander effect of ART. Although, the effect was more pronounced in case of NAC than catalase, this could be because of the involvement of some other ROS, possibly long lived radicals species^39, 40^. Further, It is worth mentioning that GJs also transmits cell-death signal from the dying cell to the healthy neighbouring cell, thus spreading the effect to large number of cell population^41^. Moreover, we have also found that ART completely inhibited the proliferation of the bystander cells. This could be because of the presence of H_2_O_2_ in the medium. A possible mechanism underlying our experimental findings has been portrayed in the Fig. 7. Overall, through a well-orchestrated interplay between GJ dependent as well as GJ independent action of Cx43, we have found a new perspective of the effective ART treatment in conjunction with Cx43 overexpression in MCF7 cells.

## Electronic supplementary material

Supplementary Info
