# Supplementary material for: Connexin-43 enhances tumor suppressing activity of artesunate via gap junction-dependent as well as independent pathways in human breast cancer cells
Source: Sci Rep. 2017 Aug 8;7:7580. doi: 10.1038/s41598-017-08058-y (PMC5548912; doi:10.1038/s41598-017-08058-y)
Supplement: Supplementary file 1 — Supplementary Info [file 41598_2017_8058_MOESM1_ESM.pdf]

Connexin-43 enhances tumor suppressing activity of artesunate via gap junction-dependent as well as independent pathways in human breast cancer cells

Asif Raza,[a] **Archita Ghoshal,**[a] **S. Chockalingam,**[a] **Siddhartha Sankar Ghosh**[a,b,*]

[a] Department of Biosciences and Bioengineering, Indian Institute of Technology Guwahati, Guwahati-39, Assam, India

[b] Centre for Nanotechnology, Indian Institute of Technology Guwahati, Guwahati-39, Assam, India

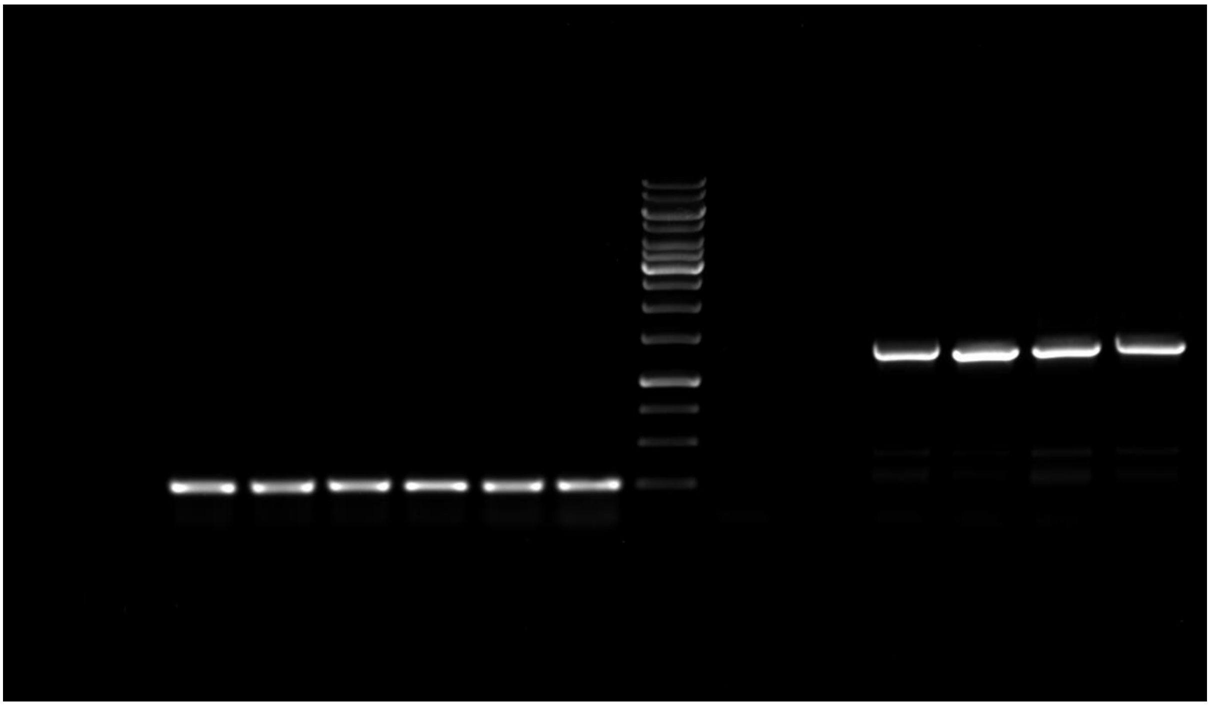

Figure 1. Uncropped gel image of figure 1 A. cDNA isolated at different time intervals and ran in the same gel.

Primers

1. CX43-Realtime1-F 5`- TCAAGCCTACTCAACTGCTGG -3`
2. CX43-Realtime1-R 5`- TGTTACAACGAAAGGCAGACTG -3`

DNA fragmentation assay

A simple method of agarose gel electrophoresis was used to examine DNA laddering due to DNA damage [1]. Briefly, viable and apoptotic cells were pelleted at 1000g for 5 min and resuspended in TES lysis buffer (100 mM Tris pH 8.0, 20 mM EDTA, 0.8% SDS). After the overnight treatment with proteinase K at 50º C, RNase was added in the lysate and incubated for 2 h at 37º C. DNA fragments were then separated by 1.5% agarose gel electrophoresis.

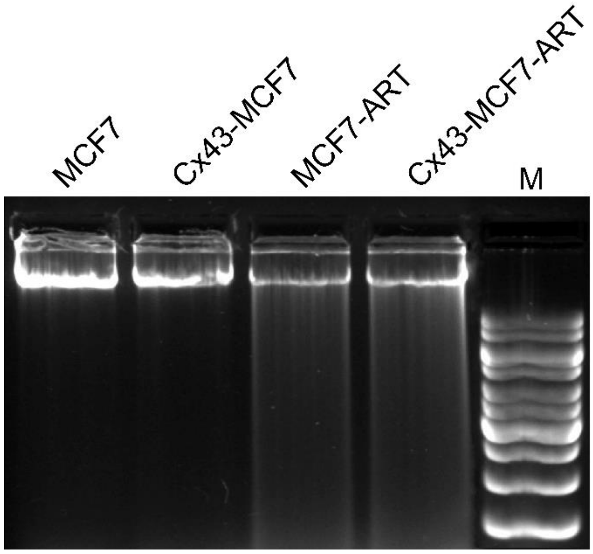

Figure 2. DNA fragmentation assay.

Densitometric analyses of Western blot images

The Western blot images of the DNA damage response pathway and GJ independent mechanism pathway were subjected to densiometric analysis using ImageJ v1.51 software. Data shown here are the ratio of protein vs respective β-actin blot.

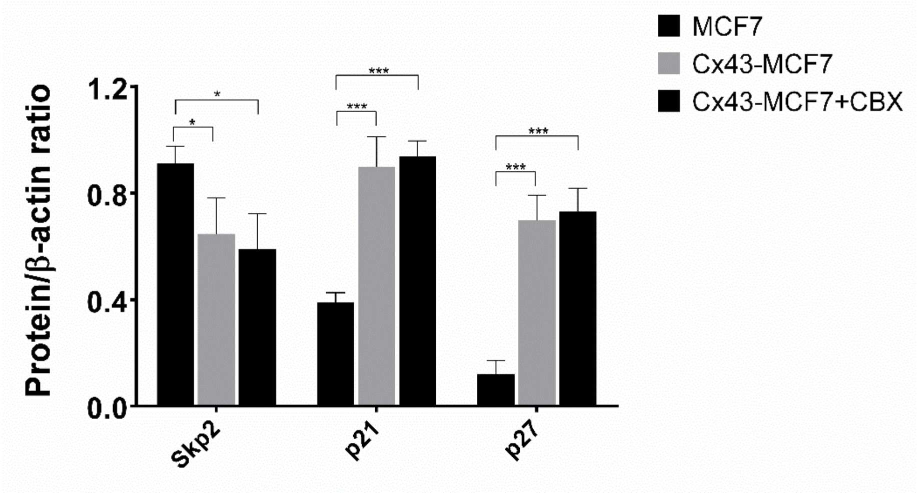

Figure 3. The band intensity of the GJ independent pathway proteins were quantitated using ImageJ v1.51 software. Data shown here as Skp2/ β-actin ratio, p21/ β-actin ratio, and p27/ β-actin ratio (n = 3).

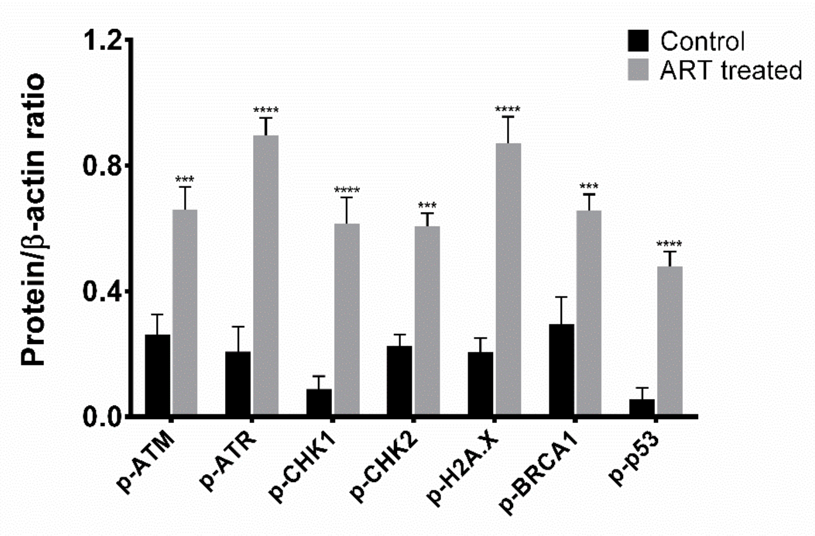

Figure 4. The relative quantification of the Western blot images of the proteins involved in the DNA damage pathway were assessed by densitometric analysis (n = 3). The data shown here are the ratio of protein/ respective β-actin intensity.

Western blot full images:

1. Beta Actin

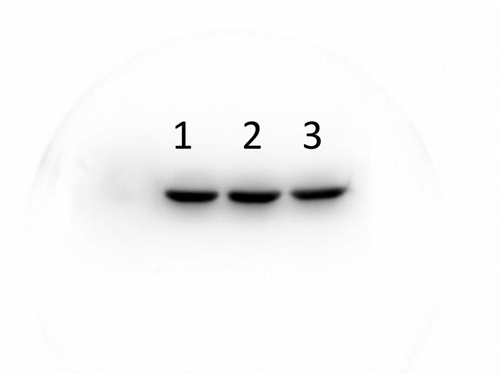

1 = MCF7

2 = Cx43-MCF7

3 = Cx43-MCF7+ CBX

2. Skp2

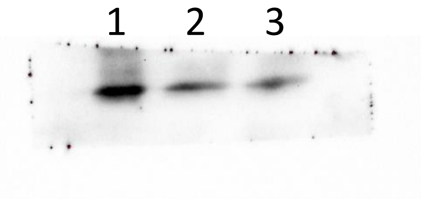

1 = MCF7

2 = Cx43-MCF7

3 = Cx43-MCF7+ CBX

3. p27

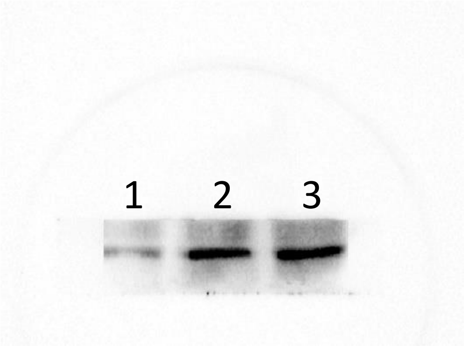

1 = MCF7

2 = Cx43-MCF7

3 = Cx43-MCF7+ CBX

4. p21

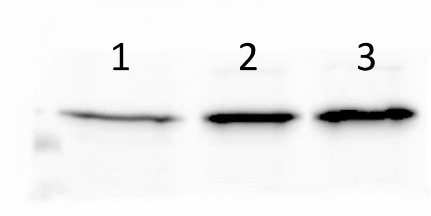

1 = MCF7

2 = Cx43-MCF7

3 = Cx43-MCF7+ CBX

4. Cx43

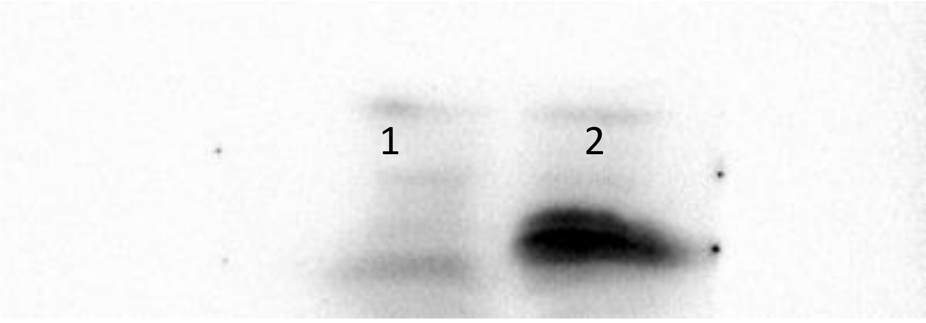

1 = MCF7

2 = Cx43-MCF7

1 Kasibhatla, S. *et al.* Analysis of DNA fragmentation using agarose gel electrophoresis. *Cold Spring Harbor Protocols* **2006**, pdb. prot4429 (2006).
